# Theory about the Embryo Cryo‐Treatment

**DOI:** 10.1002/rmb2.12027

**Published:** 2017-04-05

**Authors:** Iavor K. Vladimirov, Desislava Tacheva, Antonio Diez

**Affiliations:** ^1^ In vitro Fertilization Unit Sofia Hospital of Obstetrics, Gynaecology and Reproductive Medicine Sofia Bulgaria; ^2^ Faculty of Biology Sofia University “St. Kliment Ohridski” Sofia Bulgaria; ^3^ IGENOMIX Valencia Spain; ^4^ Infertility Institute INCLIVA Biomedical Research Valencia Spain; ^5^ Department of Obstetrics and Gynecology Valencia University Valencia Spain

**Keywords:** cryopreservation, embryo, embryo transfer, mitochondria, pregnancy

## Abstract

**Background:**

To create hypothesis, which can give a logical explanation related to the benefits of freezing/thawing embryos. Cryopreservation is not only a technology used for storing embryos, but also a method of embryo treatment that can potentially improve the success rate in infertile couples.

**Methods:**

From the analysis of multiple results in assisted reproductive technology, which have no satisfactory explanation to date, we found evidence to support a ‘therapeutic’ effect of the freezing/thawing of embryos on the process of recovery of the embryo and its subsequent implantation.

**Results:**

Freezing/thawing is a way to activate the endogenous survival and repair responses in preimplantation embryos. Several molecular mechanisms can explain the higher success rate of ET using thawed embryos compared to fresh ET in women of advanced reproductive age, the higher miscarriage rate in cases of thawed blastocyst ET compared to thawed ET at early cleavage embryo, and the higher perinatal parameters of born children after thawed ET. Embryo thawing induces a stress. Controlled stress is not necessarily detrimental, because it generates a phenomenon that is counteracted by several known biological responses aimed to repair mitochondrial damage of membrane and protein misfolding. The term for favorable biological responses to low exposures to stress is called hormesis.

**Conclusions:**

This thesis will summarize the role of cryopreservation in the activation of a hormetic response, preserving the mitochondrial function, improving survival, and having an impact on the process of implantation, miscarriage, and the development of pregnancy.

## 1 Introduction

In the last 30 years, assisted reproductive technologies have become among the fastest‐growing in medicine. They are the main method of fertility treatment, as the treatment success rate of these methods continuously increases. The reason for this is the implementation of new technologies and developments of the already‐established ones. A good example is the freezing of reproductive cells and embryos. Embryo cryopreservation and its value have grown over recent decades, thanks to the technological ability to freeze cleavage‐stage and blastocyst‐stage embryos. The first successful cryopreservation was done through using slow‐freezing methods, which have been used by specialists for the past two decades, as well. More optimized vitrification methods have been used recently, including cryo‐protectant and rapid freezing. If vitrified correctly, embryos have an outstanding survival rate of ~95%.^1^ If the development of cryopreservation technologies is compared over the years, it would be found that for a period of 10 years (from 2004 to 2013), the number of autologous frozen‐thawed embryo transfers (ETs) that were reported to the Society for Assisted Reproductive Technology (SART) increased by >2.5‐fold:^2^ in 2004, there were 15,474 frozen‐thawed ETs, compared to 40 015 in 2013, whereas the number of fresh ETs that were performed was about the same (87 089 vs 87 045). This increased use of frozen embryo transfers (FETs) corresponded with a more rapid increase in live birth rates than with fresh ETs. In 2004, the reported average live birth rate per transfer was 27.8% with FET and 33% with fresh ETs. By 2013, those average rates were 40.1% and 36.3% with FET and fresh ET, respectively. This year, clinics reported a 44.3% better delivery rate with FET, compared to 2004. The results of fresh ET only improved in those 10 years by an average of 9.8%. The results show a significant increase in the delivery rate in recent years after the cryopreservation of embryos, which is related to improved methodology by implementing vitrification. However, the methylation profiles of the children that are born after thawed ET is similar to the ones after intrauterine insemination, compared to those after fresh ET. According to the authors,^3^ the thawing of embryos has a mitigating effect on some epigenetic aberrations as a result of the in vitro fertilization (IVF)/intracytoplasmic sperm injection procedure.

Also, more and more doctors use FET to improve the success rate of the treatment of infertility. Analyzing these results, the question arises as to why FET has a higher average delivery rate, compared to fresh ET? Why is it that with the advancing age of patients, this difference increases more?^2^ Why are there higher miscarriage rates after the ET of thawed blastocysts, compared to thawed early‐cleavage embryos?^4^ What is the reason that children born after FET have healthier babies and fewer complications than those who have been implanted with fresh embryos?^5^

On analyzing some results in assisted reproductive technology, which to date have no satisfactory explanation, evidence was found to support a “therapeutic” effect of the procedure of the freezing and thawing of embryos on the recovery of the embryo and its subsequent implantation.

## 2 Freezing is Not Just Freezing

There are two basic processes of cell cryopreservation; namely, freezing and thawing. The process of freezing consists of different steps and procedures that start with the successful placement of the embryo in different media of increasing osmolarity. The loss of water (dehydration) from the embryo that results from the external increased osmotic pressure leads to, among other things, a drop in the volume of the cell. After very fast dehydration, the embryo is then placed into liquid nitrogen by using the different methods of slow freezing or vitrification. Similarly, the process of thawing consists of a variety of steps. The first step is warming up the cell from the liquid nitrogen to room temperature. Rehydration then occurs in steps, during which the embryo is moved into samples of media with decreased osmolarity and a lower concentration of cryo‐protectants. Each time, the volume of the embryo will increase because of water absorption. It will finally return to its normal size because of the concurrent outflow of cryo‐protectant. After rehydration, the embryo is placed in a normal culture medium to continue growing.^6^

## 3 What is the Effect of Freezing and Thawing on the Embryo?

Multiple intracellular and extracellular events occur in animal cells during the process of freezing. First of all, cooling to 0°C slows down cellular metabolism, disrupting active transport and ionic pumping. As a result of this, the freezing medium is osmotically balanced to reduce damage. From 0°C to −20°C, ice crystals form in the extracellular environment that increases the solute concentration of the culture medium. Water begins to move out of the cells, beginning the process of cellular dehydration and shrinkage. If the cooling process is rapid, intracellular ice crystals form before complete cellular dehydration has occurred, disrupting cellular organelles and membranes, which will affect recovery during the thawing process. When the cooling process is slow, water is osmotically pulled from the cells, resulting in improved cellular dehydration and shrinkage. Logically, the physical stress of cellular shrinking causes some damage, resulting in membrane loss and cytoskeletal and organelle disruption. Damage also can be caused by the high concentrations of solutes in the remaining unfrozen extracellular medium, resulting in membrane damage, pH shifts, and general protein denaturation.

It was found out that freezing mitochondria without any cryo‐protective agent destroys their structural integrity and functional viability and that the use of such agents prevents most, but not all, damage.^7^ The frozen embryos that maintained their quality as grade I or II were similar to those in the control group of fresh embryos. However, some ultrastructural changes, such as reduced contact between the microvilli and the zona pellucida in the blastocysts, fewer visible desmosomes, organelle‐free cytoplasmic areas, and mitochondrial swelling were observed. No rupture of the mitochondrial membranes was seen.^8^

## 4 Early Embryo Adapts and Reacts to the Environment

There are multiple sources that indicate that the embryo is not a passive group of dividing cells, but rather a living organism that struggles to survive if its living conditions are unfavorable. Early embryonic development is a critical step in human life. Embryos have the ability to adapt, develop and show differences in gene expression patterns under different conditions.^9^ Multiple molecular mechanisms and pathways control early embryonic development in response to different environments.

Culturing human embryos in low oxygen levels led to the up‐regulation of the genes that are involved in cell morphogenesis, which is relevant for embryo development and blastocyst formation.^10^ Recent findings have identified that the embryo is able to react and, in situations of low energy, activate mitochondrial maturation, thus increasing the mitochondrial DNA (mtDNA) quantity. This is supported by the observation that mitochondrial alterations are associated with increased mitochondrial proliferation; therefore, the pathogenic consequence of “mitochondrial distress” is also a marked increase in mitochondrial proliferation.^11^

## 5 Effect of Freezing and Thawing in Assisted Reproductive Technology

The theory that describes the “treatment” effect of freezing the embryo may explain the higher miscarriage rate after the ET of thawed blastocysts, compared to thawed early‐cleavage embryos. A study assessed the association between miscarriage and the transfer of fresh or thawed embryos at the cleavage and blastocyst stages. The study included 52 874 pregnancies following autologous cycles for the period between 2004 and 2008 in Australia and New Zealand. Compared to the pregnancies following thawed cleavage ET, thawed blastocyst ET was associated with a 14% higher risk of miscarriage.^4^ It is well known that stress signals, if moderate, are able to inhibit multiple apoptotic pathways.^12^, ^13^ The authors suggest that, because of the detoxified effect and rising activity of the trophectodermal cells after freezing or thawing, implantation will initiate more aneuploidy blastocysts. The presence of such embryos that are not properly and genetically structured therefore will increase the rate of spontaneous abortion. In early‐cleavage embryos, some of the aneuploidy embryos with genetic defects would not reach the blastocyst stage or there would be a developmental delay. In addition, the advantageous effect of thawing would be significantly decreased and fewer aneuploidy embryos would undergo implantation.

Another question that is waiting to be answered is why do women who become pregnant with previously frozen IVF embryos tend to have healthier babies and fewer complications than those who have been implanted with fresh embryos? A review was performed of 11 published studies that contained >37 000 pregnancies in women who had had implanted either fresh or previously frozen IVF embryos in their womb.^5^ It was found out that singleton pregnancies after the transfer of frozen‐thawed embryos were associated with better perinatal outcomes, such as antepartum hemorrhage, preterm birth, small gestational age, low birthweight, and perinatal mortality. The hormone is the basic theory by which this phenomenon is explained. Leading authors suspect that frozen IVF embryos make for healthier babies because they are implanted long after the woman's ovaries have been stimulated with drugs, so the hormone levels in the womb would have had time to return to normal. This means that the embryo is implanted in a more natural environment. The increased mitochondrial activity may, however, represent another part of the answer. The process of freezing or thawing could reduce mtDNA mutations and affect not only the process of implantation, but also the formation of the placenta and the early development of embryos. The current hypothesis, stating that the physiological levels of hormones during controlled ovarian hyperstimulation are high, is not correct because the hormonal balance in the body is changed during FET by using hormonal replacement therapy.

### 5.1 What is the explanation of this phenomenon?

**Figure 1:**
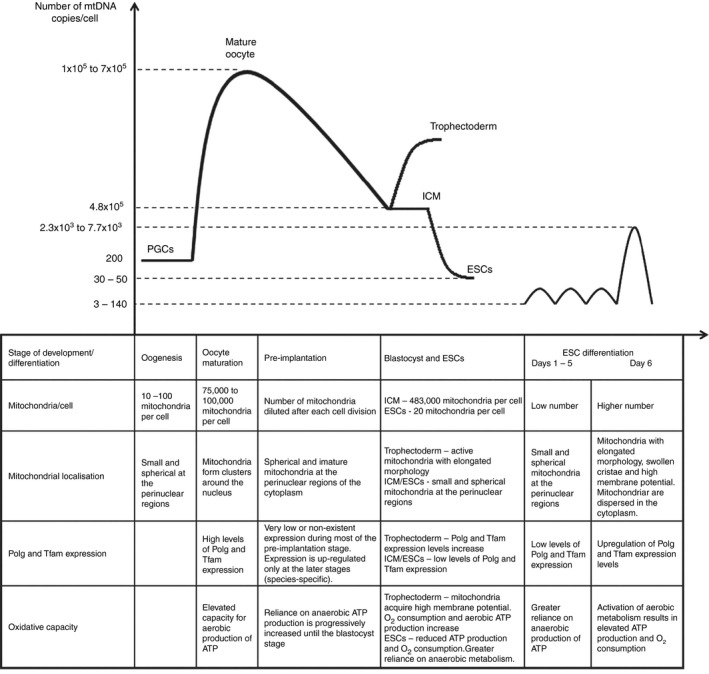
Mitochondrial biogenesis and mitochondrial DNA (mtDNA) replication.^44^ License No. 4032020938571, *Hum Reprod Update*. The replication of mtDNA is significantly reduced during pre‐implantation development, with increases only taking place in the trophectoderm in order to aid implantation. ATP, adenosine triphosphate; ESC, embryonic stem cell; ICM, inner cell mass; PGC, primordial germ cell

Another mechanism, whereby freezing and thawing has a therapeutic effect, concerns the increased mitochondrial activity that occurs during the process of embryo implantation. It has been found that, at the blastocyst stage of fresh embryos, there is an up‐regulation in the expression of mtDNA replication factors, which leads to the reactivation of mtDNA replication in the mammal species investigated thus far.^14^, ^15^, ^16^ At the same time, the mitochondria start to differentiate into elongated organelles that contain swollen cristae,^17^, ^18^ acquire a higher membrane potential and levels of oxygen consumption, and increase oxidative phosphorylation activity, leading to ATP production.^19^ However, these replication events are most likely specific to the trophectodermal cells, which give rise to the placenta and will mediate the process of implantation if the embryo is to continue to develop^20^ (Figure 1).

The procedures of freezing and thawing indicated that the oxygen consumption, which is relative to the quantity of ATP production and mitochondrial respiration of blastocysts just after warming, was significantly lower than that of the fresh blastocysts. The oxygen consumption rate of the blastocysts increased with time after warming. The blastocysts that retained high developmental competence recovered rapidly, compared to those in the other groups. Moreover, the mitochondrial cytochrome oxidase activity was not observed at 0 hour, but rather at 24 hours after warming. These data suggest that the mitochondrial functions are restrained or obstructed somewhere during the cryopreservation process. Furthermore, the oxygen uptake by vitrified bovine blastocysts, 18 hours after thawing, was the same as that of the non‐vitrified blastocysts. Another study has found that, after 6 hours, the oxygen consumption rate of the vitrified and the heated human blastocysts in the hatched group had recovered to that exhibited by the fresh blastocysts.^21^, ^22^

**Figure 2:**
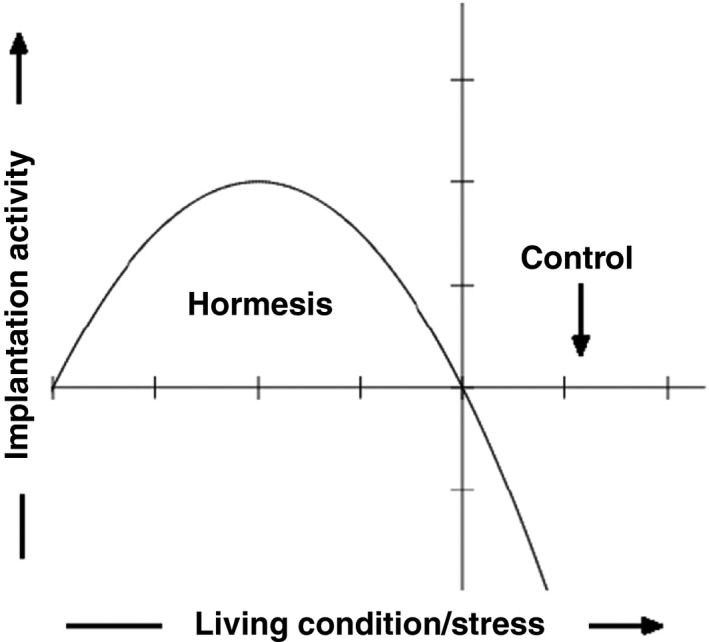
Effect of hormesis on the implantation activity of embryos during a change in the development conditions

From the studies described above, it can be concluded that there is increased mitochondrial activity after thawing, which is similar to the physiological processes occurring in trophectodermal cells. This jump in oxygen consumption after freezing and thawing probably had a positive effect in the initiation of embryo implantation. This process induces a stress. Thus, a pollutant or toxin that shows hormesis has the opposite effect in small doses, as it does in large doses (Figure 2). Hormesis is a response that induces protective signals.^21^

If the stress does not occur, that means that the protective signals are not there. Freezing and thawing generates alterations in protein structures (because of changes in the water amount and structure) and these alterations induce response signals (chaperones, anti‐oxidant responses, and protective enzymes) that are able to repair small defects that are present in the embryo during the post‐thawing period. Poor‐quality embryos might therefore derive a benefit from these stress signals. An unfolded protein response occurs in the mitochondrial and cellular environments and persists for a time after the stress has disappeared. Indeed, it has been clearly demonstrated that the activation of the unfolded protein response gives protection in multiple scenarios, like ischemia,^23^ metabolic alterations,^24^ or neurotoxins.^25^ The hormesis response during embryonic development has been reported in multiple species, such as flies,^26^, ^27^ zebrafish,^28^ duck,^29^ and mice.^30^

**Figure 3:**
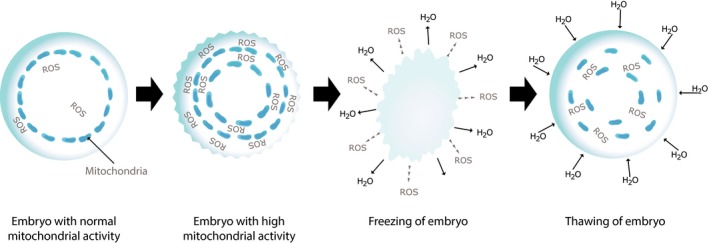
Decrease of mitochondrial activity and the levels of reactive oxygen species (ROS) in the embryo after freezing and thawing procedures

Summarizing the above theoretical hypothesis, “cryo‐treatment of the embryo” has two main components. As a result of freezing or thawing of the embryos, there are reduced levels of reactive oxygen species (ROS), detoxification of the cells is carried out, and the amount of mutated mtDNA is reduced (Figure 3). Another mechanism of influence is through the rapid recovery (jumping effect) of mitochondrial activity in the trophectodermal cells of the blastocyst, which is part of the physiological process of implantation.

### 5.2 What do the statistics tell us? How can we explain them?

By use of this theory, some of the controversial results that were derived from clinical practice can be explained. With scrupulous attention to the numerous details and proper application of the latest vitrification techniques, the efficiency of cryopreservation has been improved substantially. Live births from FETs represented 31.5% of all reported autologous live births in 2012, compared with just 16.9% in 2006.^31^ The increase in FET usage and success rates might have resulted from multiple simultaneous causes. Improved cryopreservation techniques could reduce cryo‐embryo damage and therefore increase the success rates and confidence in cryopreservation and FET. This is the policy of freezing “second‐best” embryos after the morphologically best embryos are transferred in fresh cycles. As a result of the improving FET results, the question arises as to why the policy of “freezing all” embryos has not been applied. Both issues are still controversial and the results that have been derived from FET are not statistically different, compared with those derived from fresh ET.^32^ The data that were derived from a 2013 study that reported the percentage of ETs resulting in live births, comparing the results from fresh ETs vs FETs in different age groups, is very interesting as it showed a significant increase in the live birth rate from FETs after the age of 37 years.^2^ Up to the age of 35 years, the figures were 47.7% vs 44.4%; from 35 to 37 years of age, they were 39.2% vs 40.6%; from 38 to 40 years of age, they were 28.5% vs 36.1%; from 41 to 42 years of age, they were 16.3% vs 31.6%; and for >42 years of age, they were 7.3% vs 21.2%. Other authors also discovered that, with an advancing age in women, the percentage of pregnancies was higher in the cases with thawed ET, compared to fresh ET.^33^, ^34^

Here, two questions arise. How can we explain the phenomenon that, with the advancing age of women, the delivery rate after FET is higher in comparison to fresh ET? Why, in women aged ≤35 years, are the deliveries by fresh ET higher compared to FET?

In pre‐implantation embryos, the unfolded protein response is present and has been identified by the identification of molecules, such as Atf4, Atf6, Ask1, Bip, Chop, Gadd34, Ire1, Perk, and Xbp1, which work as master regulators of this process.^8^ This indicates that pre‐implantation embryos use the unfolded protein response coping response as one mechanism to deal with pre‐implantation stress. Therefore, in mice that are lacking the unfolded protein response‐associated genes, the embryos do not survive beyond pre‐implantation. All of this indicates the important role of these responses in pre‐implantation embryos.^35^ In addition, the stress response pathways that are linked to Perk activate nuclear factor erythroid 2‐related factor 2 (NRF2). The activation of NRF2 leads to increased glutathione levels and the buffering of ROS, providing a valuable connection between the unfolded protein and oxidative stress response.^14^

**Figure 4:**
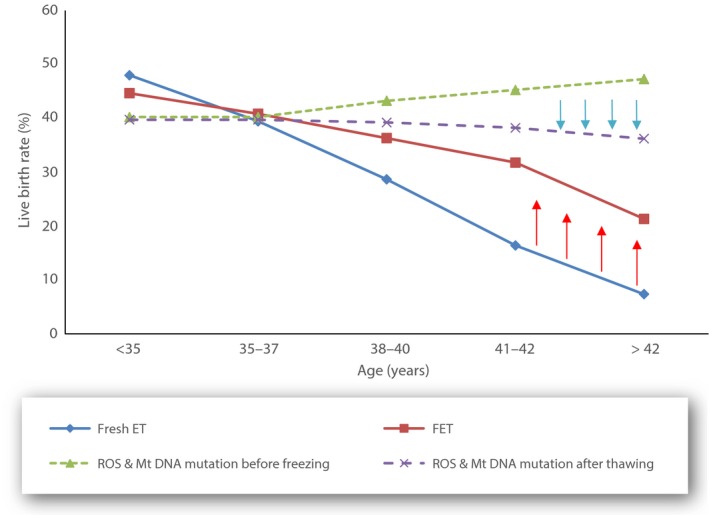
Effect that the freezing and thawing of embryos has on the levels of reactive oxygen species (ROS), mitochondrial DNA (mtDNA) mutations, and live birth rate in different ages of women. ET, embryo transfer; FET, frozen embryo transfer

On the basis of the theory described above, the freezing and thawing of embryos reduces the levels of ROS and mitochondrial activity, respectively, producing more embryos with a value that is below the threshold of mtDNA quantities that were described in one relevant study.^36^ Thus, there are more “healthy” embryos and a higher implantation potential in women of advanced maternal age (Figure 4). In support of this, another study established that, in women of advanced reproductive age, there are statistically higher values of mtDNA present.^37^ Unfortunately, at this time, the results are not sufficient to definitely confirm or reject the theory. Mitochondria in the oocytes of older hamsters and mice have been shown to generate higher levels of ROS, produce less ATP, and therefore are likely to have a reduced capacity to adequately support a dynamic process, such as pre‐implantation development.^38^ Oocyte growth and nourishment are supported by the surrounding granulosa cells and a deterioration in the characteristics of these granulosa cells that are derived from full‐grown antral follicles have been observed in aged women. In addition, a decline in ATP synthetic capability with age could be related to an accumulation of mutations in the granulosa mitochondrial DNA.^39^ This deficiency in granulose energetic production consequently leads to a reduced energetic reserve in the oocyte. A reduced energetic reserve is a signal that induces an increase in the mitochondrial number. This increment might be necessary in the embryos of older women in order for sufficient ATP levels to be maintained for normal development. However, the location of the mtDNA in close proximity to the ROS generated by the respiratory chain, coupled with a lack of histones and inferior DNA repair mechanisms, leaves the mitochondrial genome particularly vulnerable to mutation. Thus, this increment in oocyte mitochondrial activity in order to solve the reduced energetic reserves would lead, as a consequence, to an increase in the mtDNA mutations in the oocyte. This increase in mtDNA mutations would increase the mtDNA copy number again. An increase in the mtDNA content of human pre‐implantation embryos in response to mutation has been documented previously.^11^

The answer to the second question is related to the suggestion of a lower limit of mitochondrial activity of the embryo, under which implantation cannot occur. According to the hypothesis about embryo cryo‐treatment, the procedure of freezing and thawing causes a reduction of mitochondrial activity below the minimal threshold that is needed for an implantation to occur. With this assumption about the minimum threshold, the lower rate of implantation and births with their own eggs in thawed ET, compared to in fresh ET in women aged ≤35 years old, may be explained. This hypothesis about the existence of a minimum threshold of mitochondrial activity explains the higher success rate in fresh ET, compared to thawed ET, in the donor program (49.6% to 37.5%, respectively), as well. The explanation of a lower success rate in thawed ET is that the egg donors are mainly women aged ≤35 years old, where a reduction of normal mitochondrial activity through freezing and thawing causes mitochondrial levels to fall below the corresponding minimum threshold, which leads to a reduction in the implantation opportunities of the generated embryos.

**Figure 5:**
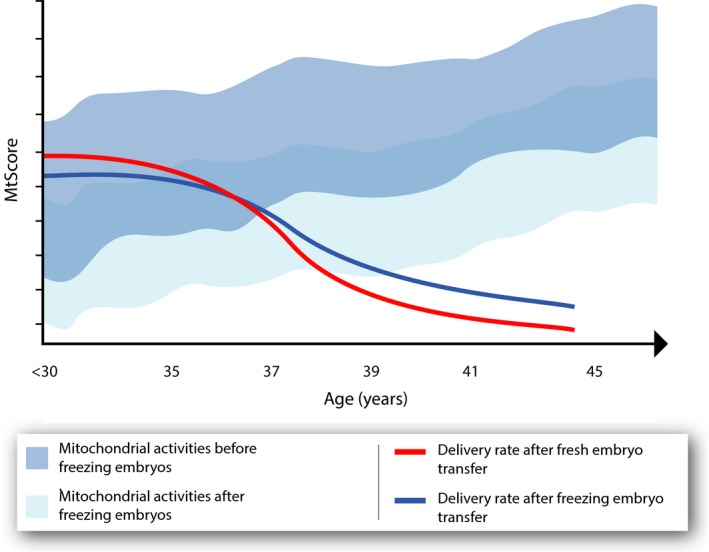
Effect of embryo freezing and thawing on the displacement of the mitochondrial levels in different ages of women and how it is related to the delivery rate. mtScore, method of assessing the level of mitochondrial activity

In the above context, it is logical that, in this situation, the policy of freezing all embryos is not suitable in women who are younger than 35 years and it is recommended to carry out fresh ETs wherever possible. As women age, the decrease in mitochondrial activity to below the minimal threshold that is associated with implantation after freezing and thawing has another clinical effect. This occurs mostly in women who are older than 37 years. In embryos with a higher mitochondrial activity due to a lack of implantation, the cryo‐procedure causes a suppression of the activity to levels that allow the implantation of embryos^2^ (Figure 5).

Finally, although it is commonly believed that the mitochondria support embryonic development from early cleavage until the blastocyst stage,^40^ several studies have demonstrated that this is not the case, at least in animal models. Mice, deficient in mitochondrial transcription factor A, have a reduced mitochondrial number in the oocytes, but fertilization or early embryonic development is not affected. In addition, the deletion of essential genes for the preservation, replication, and expression of mtDNA, although resulting in embryonic lethality, did not show changes in the embryo implantation rates.^41^ Also, from a biological standpoint, it is logical that, if the oocyte and early embryo are dependent on external metabolic sources, the embryos with an increased capacity for nutrient and oxygen exchange evolution would have a competitive advantage, but the oocytes and early embryos are almost spheres, which is the geometrical form with a lower surface area and therefore less exchange capacity. These observations suggest that the energy that is necessary for embryonic development is provided up to a threshold by the accumulated reserves that are present in the oocyte and only in extreme cases of reduced metabolic fuel will the cell machinery react to increase the mtDNA copy number in an attempt to produce more mitochondria that are not fully functional.

It is known that mitochondrial activity is essential in human reproduction. The presence of mitochondrial dysfunction secondary to mtDNA deletions is tissue‐specific and has been linked to many pathologies, such as diabetes, heart failure, neurodegenerative diseases, and even cancer.^42^ However, as mtDNA mutations affect only a subset of organelles, usually ≥60% of the mtDNA copies need to contain deletions before any significant biochemical defect would be apparent. As the accumulation of mutations in the mtDNA is often blamed for reproductive decline with age, some studies have tried to determine the connection between mtDNA mutations and reproductive performance.^43^ In addition, the offspring of older mothers (≥40 years of age) have been observed to have a significantly higher rate of male infertility because of asthenozoospermia and female subfertility that is associated with oligomenorrhoea and amenorrhoea, probably representing polycystic ovary syndrome, both conditions that are associated with mitochondrial dysfunction. Diabetes, Alzheimer's disease, and metabolic syndrome are examples of other common problems that are now regarded as diseases with mitochondrial involvement.^44^

## 6 Conclusion

In conclusion, the authors believe that the early embryo in its natural environment is relatively independent of external metabolic and energetic supplementation. Furthermore, the embryo is able to adapt, as demonstrated by in vitro cultured embryos adapting to new conditions and developing normally. This ability of adaptation allows the embryo to respond to different microenvironmental conditions. The embryos are able to increase mitochondrial DNA, probably in order to try to solve energy demands, and although this is not sufficient to solve all their energy needs, this increase of mtDNA can be used as a marker of embryos with poor implantation potential.^2^ The theory of “washing” and the hormesis response of embryos after freezing and thawing are concepts that have to be explored. The unfolded protein response has been implicated in lifespan extension in worms, flies, and a mouse, suggesting a conserved role in the long‐term maintenance of cellular homeostasis, and this probably is contributing to the better implantation rates of thawed embryos. In contrast, the possibility of the advantages arising from the freezing and thawing process described here should enable researchers to rethink the position of cryobiology in reproductive medicine.

## Disclosures

*Conflict of interest*: The authors declare no conflict of interest. *Human and Animal Rights*: This article does not contain any study with human or animal participants that have been performed by any of the authors.
